# Global, regional, and national burden of falls from 1990 to 2021: a comprehensive analysis with a focus on falls attributable to reduced bone mass and projections to 2045

**DOI:** 10.3389/fpubh.2025.1695026

**Published:** 2025-11-11

**Authors:** Yi Liu, Junjie Qiu, Renlin Huang, Yong Zhou, Ziqiang Huang

**Affiliations:** 1 Orthopedics Department, Ziyang People's Hospital, Ziyang, Sichuan, China; 2 West China Second Hospital, Sichuan University, Chengdu, Sichuan, China

**Keywords:** falls, reduced bone mass, incidence, prevalence, deaths, DALYs, projection

## Abstract

**Background:**

This study aims to analyze the global epidemiological trends of falls from 1990 to 2021, providing an in-depth understanding of their evolving patterns. It explores the changing trends of falls caused by reduced bone mass in middle-aged and older adults, offering valuable insights to healthcare policymakers for more effective resource allocation.

**Methods:**

Data from the Global Burden of Disease Study 2021 (GBD 2021) were used to comprehensively assess the global incidence, prevalence, mortality, and disability-adjusted life years (DALYs) associated with falls. Regions were categorized using the Socio-demographic Index (SDI), and the correlation between the fall burden and economic level was evaluated. Additionally, long-term trends were estimated using Joinpoint regression analysis, and the norpred prediction model was employed to assess future disease burden trends.

**Results:**

From 1990 to 2021, the age-standardized incidence rate (ASIR), age-standardized prevalence rate (ASPR), age-standardized death rate (ASDR), and DALYs related to falls showed a global downward trend, although the relative number of cases continued to rise. In 1990, the ASIR was lowest in low SDI regions, while the ASDR was at an intermediate level. By 2021, the ASIR remained the lowest in low SDI regions, but the ASDR reached its highest point. From a national and regional perspective, the number of fall-related cases in 2021 almost doubled compared to 2019. The global burden of falls increased with population aging, and the disease burden is projected to continue growing over the next few decades. Analysis of falls attributable to reduced bone mass found that women's DALYs were higher than men's after the age of 50, and there was a general decreasing trend in DALYs for the 40–59 age group, followed by an increase after 2019. Decomposition analysis indicated that population growth and aging were the main factors driving the increase in incidence and mortality.

**Conclusion:**

Although the ASIR of falls has decreased annually, the global burden continues to rise due to population growth and aging. Lack of physical activity is also a contributing factor to falls associated with reduced bone mass. Tailored prevention and treatment strategies for middle-aged and older adults are essential to reduce this burden and improve patient outcomes.

## 1 Background

Unintentional falls represent a major global cause of injury, accounting for nearly 700,000 deaths each year and ranking as the second leading cause of fatal unintentional injuries after road traffic accidents (1). In 2015, fall-related medical costs in the United States alone were estimated to approach USD 50 billion (2). Falls are particularly significant in older adults, being a predominant external cause of disability, functional impairment, and death, and are associated with increased risks of severe trauma, hospitalization, and mortality (3).

Epidemiological data indicate that nearly one-third of community-dwelling individuals aged 65 and older experience at least one fall annually, with approximately half experiencing recurrent falls (4). The etiology of falls is multifactorial, including cognitive decline (5), unsafe home environments (6, 7), and polypharmacy (8). Late-life falls are associated with a sharp decline in overall health status (9), particularly when coupled with decreased bone mass. In such cases, the risk of fall-induced fractures—especially at the hip, spine, and forearm—increases substantially, often resulting in greater injury severity, prolonged rehabilitation, and elevated likelihood of long-term disability or death (10). Reduced bone mass is frequently accompanied by loss of muscle mass, a syndrome referred to as osteosarcopenia. Sarcopenia contributes to gait instability and slowed neuromuscular response, directly increasing fall risk.

Although the most severe consequences of bone-related falls are observed in older adults, recent trends suggest a growing burden among middle-aged populations as well. Studies have shown that hospitalization rates for fall-related injuries have been steadily increasing among individuals aged ≥10 years (3). Given the substantial geographic, temporal, and age-related variations in the epidemiology of falls (11), a comprehensive global analysis of fall trends—particularly those associated with low bone mass—in middle-aged and older adults is essential to inform evidence-based public health strategies and optimize the allocation of healthcare resources.

The Global Burden of Disease (GBD) database is an ongoing, large-scale international initiative designed to quantify health loss across the world. It provides comprehensive estimates of disease burden attributable to 88 risk factors across 204 countries and 811 subnational locations (12). Despite the release of updated GBD data in 2021, no comprehensive global-level analysis has yet been conducted focusing specifically on the burden of falls—particularly those associated with reduced bone mass—in middle-aged and older adults. The objective of this study is to systematically evaluate the trends in disease burden due to unintentional falls among the middle-aged and older adults worldwide. By analyzing data from the GBD 2021 update, the study aims to uncover the global distribution and magnitude of fall-related health losses and explore their association with low bone mass as a contributing risk factor.

At the same time, by evaluating the temporal trends in disability-adjusted life years (DALYs) attributable to low bone mass-related falls among individuals aged 40–59 years, stratified by sex, age group, and Socio-demographic Index (SDI) levels, this study clarifies the burden in a population often overlooked in fall-related research. The rationale for focusing on this age group is to identify non-aging determinants of skeletal fragility and associated injury burden—factors that are frequently underrepresented in current fall-prevention frameworks that are predominantly centered on older adults. By analyzing these data, the aim is to reveal the distribution and severity of falls worldwide. The results of this study will highlight regions with heavy disease burdens and help develop targeted prevention and control guidelines for countries and regions.

## 2 Methods

### 2.1 Data collection

All data analyzed in this study were obtained from the Global Burden of Disease Study 2021 (GBD 2021), developed and maintained by the Institute for Health Metrics and Evaluation (IHME) at the University of Washington. The GBD 2021 provides comprehensive and comparable estimates of disease burden, including mortality, morbidity, and risk factors, across global, regional, and national levels.

### 2.2 SDI

The global socio-demographic index (SDI) was included in this study to explore the relationship between trends in adverse effects of medical treatment and levels of development. Countries were categorized into five SDI groups: low SDI (SDI <0.46), low-moderate SDI (0.46–0.64), medium SDI (0.65–0.74), high-moderate SDI (0.75–0.85), and high SDI (SDI > 0.85) (13).

### 2.3 Statistical analysis

Age-standardized rates (ASRs) for incidence, prevalence, deaths, and disability-adjusted life years (DALYs) associated with falls, as well as DALYs attributable to low bone mineral content as a risk factor for falls, were obtained. ASRs were used to facilitate cross-regional comparisons and to account for variations in population size and age structure.

To evaluate temporal epidemiological trends, Joinpoint regression analysis was performed using the Joinpoint Regression Program. Specifically, a log-linear model was fitted to the ASRs, assuming that the rates changed at a constant percentage per year within each segment. The number of joinpoints was automatically determined by the permutation test method, and model selection was based on the Bayesian Information Criterion. The minimum number of observations from a joinpoint to either end of the data series, as well as between joinpoints, was set to two, following the default configuration. The average annual percentage change (AAPC) and the corresponding 95% UI were calculated from the fitted model to quantify temporal trends.

Data from 1990 to 2021 on fall-related incidence, prevalence, deaths, and DALYs were included as input variables. The standard error of the estimates was computed using the following formula:


$$\begin{array}{l} \text{SE}=\left(\text{upper}-\text{lower}\right)/\left(\text{1}.\text{96}\times \text{2}\right) \end{array}$$

where “upper” and “lower” represent the bounds of the 95% UI provided in the GBD dataset. Geometric weighting was applied to calculate the AAPC and its corresponding 95% UI for each region, thereby characterizing temporal trends in incidence, prevalence, deaths, and DALYs over the 32-year period (1990–2021).

Future trends in the fall-related burden were projected using the “nordpred” package in R, which implements an age–period–cohort (APC) model based on Poisson regression. Observed data from 1990 to 2021 served as the input period. The model automatically divided the data into 5-year age and period intervals and projected incidence through 2045 while accounting for recent trend attenuation. A power5 link function was used instead of the conventional log link to prevent overestimation of future trends.

Statistical significance was defined as *P* < 0.05. All analyses were conducted using the Joinpoint Regression Program (version 2024) (14) and R software (version 4.3.0).

## 3 Results

### 3.1 Global trends

Between 1990 and 2021, the global number of fall-related cases increased markedly from 326.90 million (95% UI: 280.68–382.95 million) to 540.88 million (95% UI: 472.96–615.11 million). Despite this substantial increase in absolute numbers, the age-standardized rate (ASR) of fall cases declined from 7,279.6 per 100,000 (95% UI: 6,310.5–8,385.2) to 6,455.4 per 100,000 (95% UI: 5,652.6–7,337.1) over the same period. Similarly, the number of incident cases rose from 155.95 million (95% UI: 136.20–179.72 million) in 1990 to 215.57 million (95% UI: 195.27–238.98 million) in 2021. However, the ASR for incidence declined from 3,002.3 (95% UI: 2,665.3–3,411.4) to 2,702.0 (95% UI: 2,444.5–3,000.4).

In terms of fall-related mortality, the number of deaths nearly doubled, from 407,768 (95% UI: 357,240–450,140) in 1990 to 802,803 (95% UI: 681,874–874,338) in 2021. Correspondingly, the mortality ASR slightly decreased from 10.9 per 100,000 (95% UI: 9.7–11.8) to 9.9 per 100,000 (95% UI: 8.4–10.8). The DALYs attributable to falls increased from 29.41 million (95% UI: 24.56–35.19 million) to 43.80 million (95% UI: 35.94–52.81 million), while the DALY ASR declined from 643.1 per 100,000 (95% UI: 539.5–767.6) to 531.3 per 100,000 (95% UI: 436.9–639.1). This suggesting improvements in age-specific rates despite a growing and aging global population (Supplementary Table 1).

Further analysis using annual percentage change (APC) and average annual percentage change (AAPC) revealed distinct temporal patterns. The most significant decline in incidence ASR was observed between 2000 and 2005 with an APC of −1.23%, whereas a modest increase occurred during 2018–2021 (APC = +0.78%). The overall AAPC for incidence was −0.32%.

**Figure 1:**
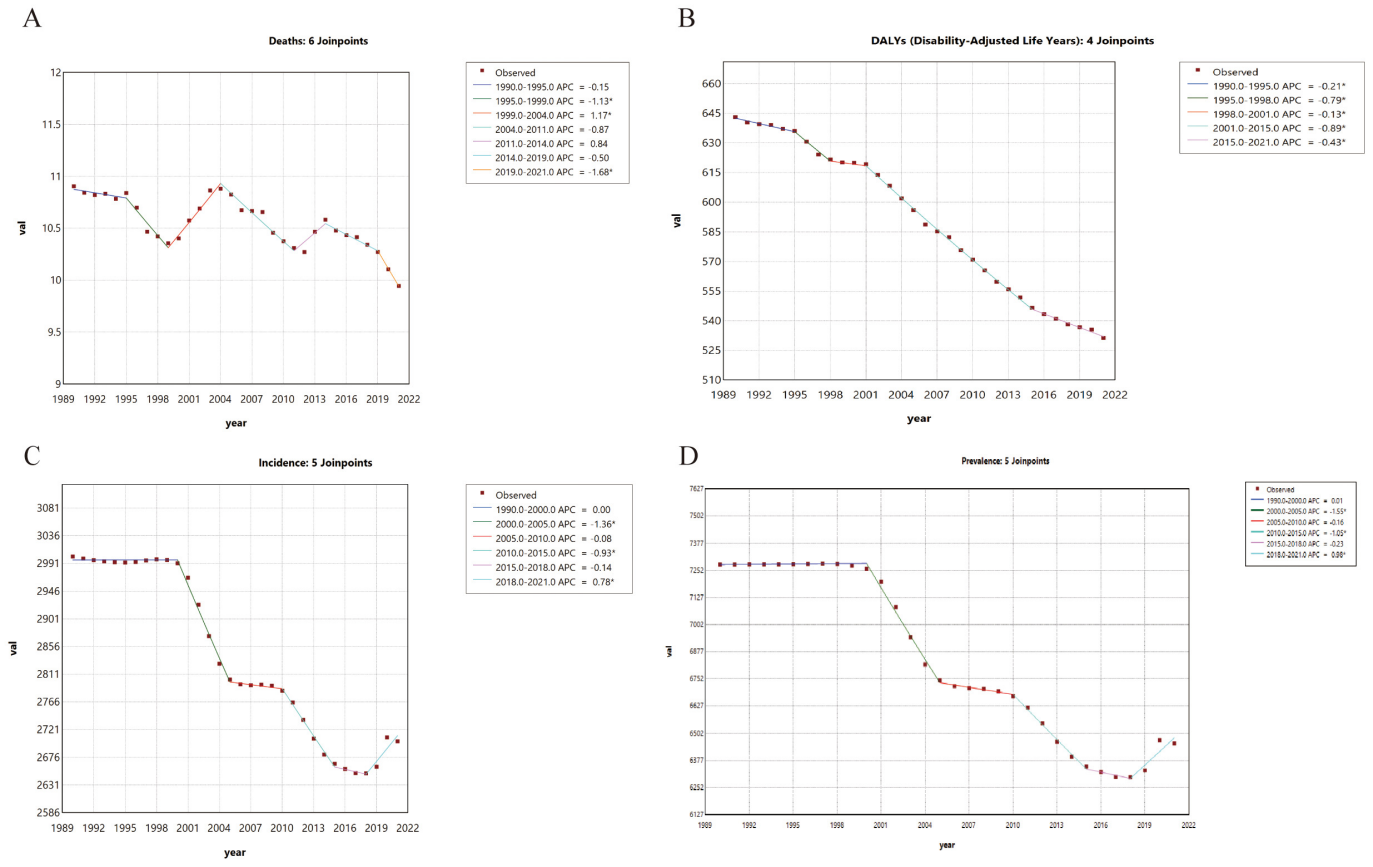
Joinpoint Regression Analysis of the Global Burden of Falls, 1990–2021. **(A)** Trends and annual percentage changes (APC) in fall-related mortality. **(B)** Trends in disability-adjusted life years (DALYs) due to falls. **(C)** Trends in fall incidence. **(D)** Trends in fall prevalence. Each joinpoint represents a time node indicating a significant change in trend, and the lines denote the APC for each segment. An asterisk (*) indicates statistical significance (p <0.05).

The prevalence ASR showed a similar pattern, with the largest decline between 2000 and 2005 (APC = −1.56%) and a minor increase during 2018–2021 (APC = +0.98%), resulting in an overall AAPC of −0.37%. Furthermore, Mortality ASR demonstrated a fluctuating downward trend, with an overall AAPC of −0.29%. Likewise, DALY ASR followed a similar trajectory, exhibiting a wavelike decline with an overall AAPC of −0.61% (Figure 1).

### 3.2 Regional trends

In 2021, studies conducted at the global regional level showed that the age-standardized prevalence rate (ASPR) of falls was highest in high SDI regions, with 9,086.6 (95% UI: 7,948.3–10,367.3) cases per 100,000 people (Supplementary Table 1), while low SDI regions had an ASPR of 3,473.9 (95% UI: 3,055.8–3,954.5) cases per 100,000 people. Geographically, Australasia had the highest ASPR, with 12,862.8 (95% UI: 10,938.8–15,735.4) cases per 100,000 people, while Africa generally exhibited lower rates, with Southern Sub-Saharan Africa having the lowest rate of 1,920.3 (95% UI: 1,648.2–2,202.5) cases per 100,000 people. Between 1990 and 2021, changes in ASPR varied across different regions of the world. The high-middle SDI regions observed the most significant decline, with an ASPR of 7,952.3 (95% UI: 6,888.2–9,131.7) cases per 100,000 people in 2021, while low SDI regions showed the least decline, with an ASPR of 3,473.9 (95% UI: 3,055.8–3,954.5) cases per 100,000 people in 2021. Other SDI regions exhibited slight declines.

### 3.3 SDI and fall burden

**Figure 2:**
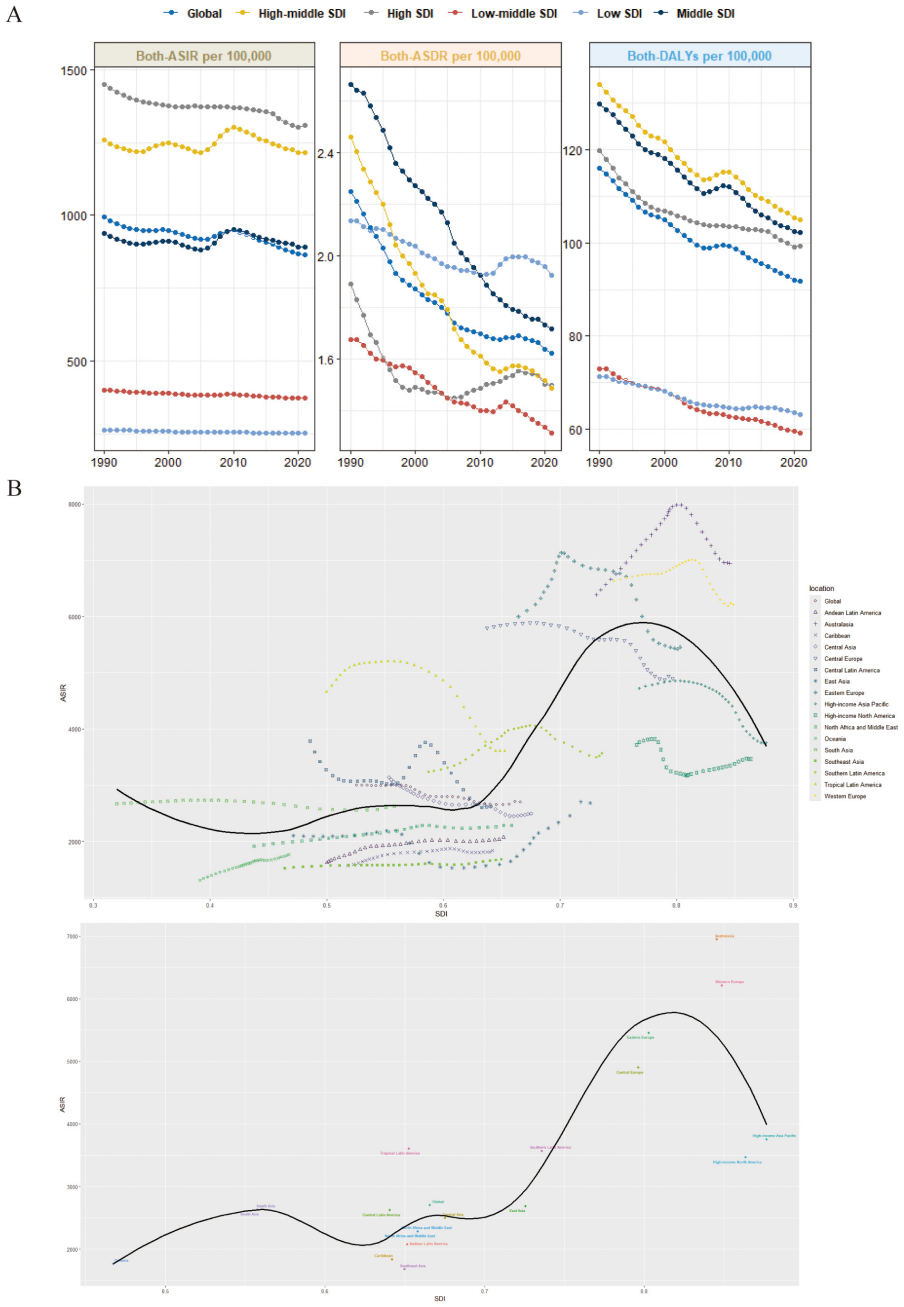
Global burden of falls. **(A)** Trends in age-standardized incidence rates, age-standardized mortality rates, and disability-adjusted life years (DALYs) for falls from 1990 to 2021 across different Social Development Indices (SDIs) (per 100,000 population). **(B)** Age-standardized incidence rates of falls for 17 Global Burden of Disease (GBD) regions.

From 1990 to 2021, low SDI regions, initially having the highest ASIR in 1990, have seen a gradual decline. However, in 2021, they still exhibited the highest ASIR globally. The ASDR showed a clear downward trend across all SDI regions, but in low SDI regions, the ASDR, which was lower than the global average in 1990, became the highest in 2021. In 2021, countries and regions with higher SDI had relatively higher ASIRs (Figure 2).

### 3.4 Country-level trends

**Figure 3:**
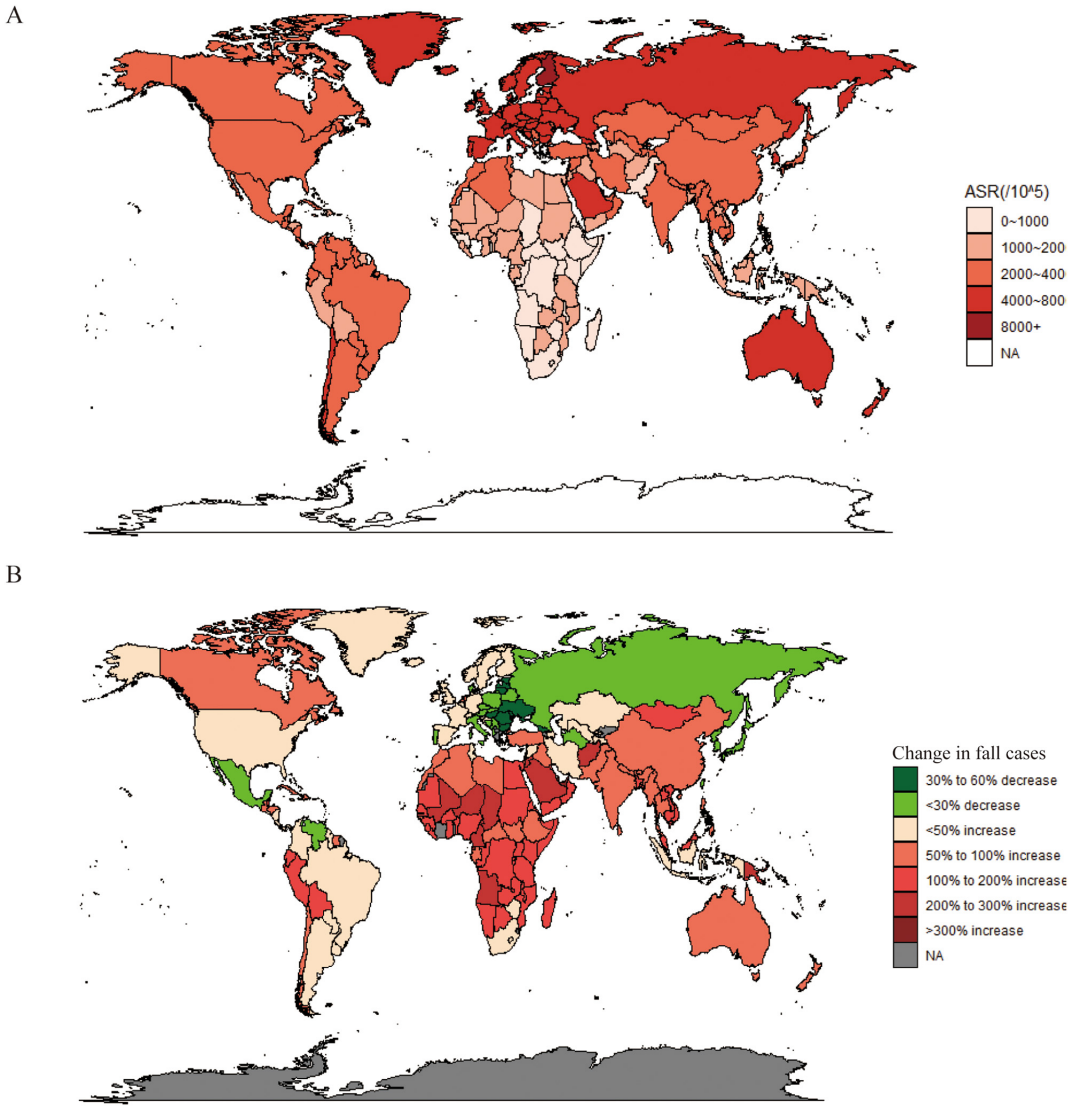
Analysis of falls in 204 countries and territories using multiple metrics. **(A)** Age-standardized incidence rate in 2021. **(B)** Change in cases from 1990 to 2021. World maps created using: (1) Spatial Visualization with ggplot2 package by David Kahle, licensed under GPL-2 and (2) Draw Geographical Maps. R package by Richard A. Becker, Allan R. Wilks, Ray Brownrigg, Thomas P. Minka and Alex Deckmyn, licensed under GPL-2.

In 2021, Andorra had the highest ASIR at 9,011.5 (95% UI: 8,131.2–10,036.6), followed by Swaziland with an ASIR of 8,069.9 (95% UI: 7,023.9–9,285.9). North Korea had the lowest ASIR at 689 (95% UI: 622.5–763) (Figure 3). Compared to 1990, the global incidence of falls in most countries showed a substantial increase, with the case numbers of several countries growing by over 200%. Qatar saw an increase of more than 600%.

### 3.5 Age patterns

**Figure 4:**
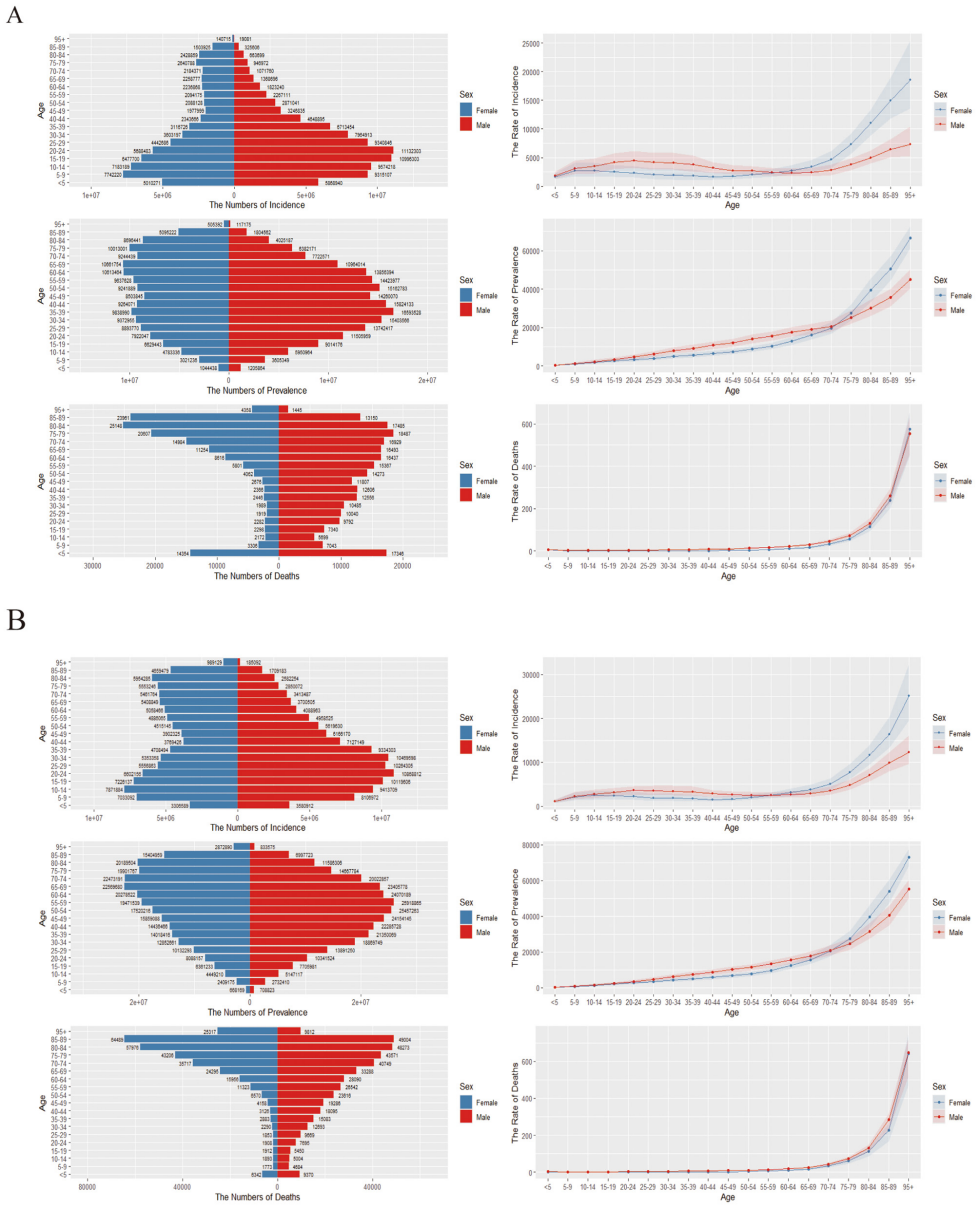
Mortality, and DALYs across different age groups. **(A)** Trends in global incidence, mortality, and DALYs due to falls in 1990. The left side shows the number of cases, while the right side displays the incidence rate. **(B)** Trends in global incidence, mortality, and DALYs due to falls in 2021. The left side indicates the number of cases, and the right side illustrates the incidence rate.

In 2021, the ASIR of falls globally increased with age, peaking in individuals aged 95 and older. The 55–59 age group served as a midpoint, with the incidence rates for men and women exhibiting an inverse relationship before and after this age. This pattern was similar to that observed in 1990 (Figure 4).

### 3.6 Falls due to reduced bone mass

**Figure 5:**
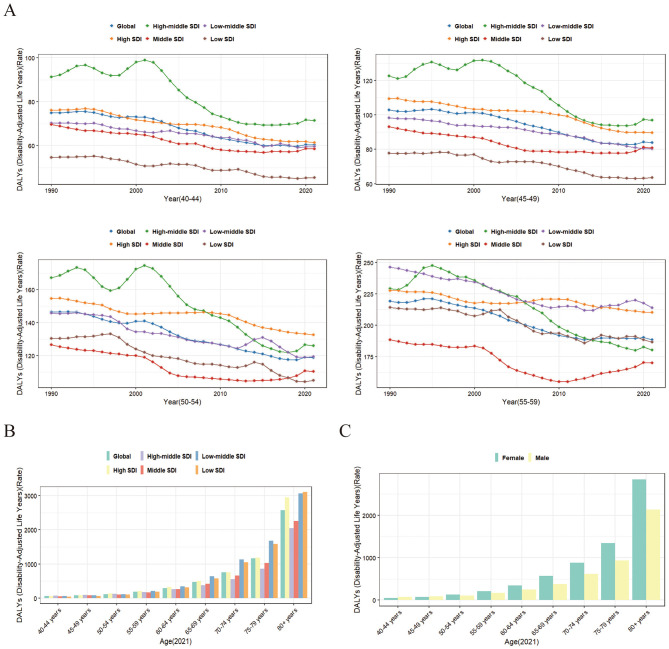
Trends in fall-related DALYs due to bone loss among middle-aged and older populations. **(A)** Trends in fall-related DALYs from bone loss in the 40-59 age group across different Social Development Indices (SDI) from 1990 to 2021. **(B)** Trends in fall-related DALYs due to bone loss among different age groups in 2021 across varying SDI. **(C)** Trends in fall-related DALYs due to bone loss among different age groups and genders in 2021.

According to 2021 statistics, DALYs attributable to falls caused by low bone mass increased with age, indicating that older individuals with osteoporosis experienced longer disability durations due to falls. In high SDI countries, the years lived with disability were higher, while middle SDI countries showed lower DALYs across multiple age groups compared to both high-middle and low SDI countries. Conversely, in middle SDI countries, the DALYs gradually exceeded those in high-middle SDI countries after the age of 60, while low SDI countries had relatively fewer disability years, likely due to higher mortality rates caused by poorer healthcare conditions (Figure 5B).

Gender-specific studies showed that in 2021, women aged 50 and above had significantly higher DALYs due to falls caused by low bone mass compared to men, with the gap widening as age increased (Figure 5C).

To further explore falls related to reduced bone mass in middle-aged individuals, we analyzed DALYs for the 40–59 age group from 1990 to 2021. The results revealed a trend of decline followed by an increase globally. Specifically, between 1990 and 2019, the global trend showed either a decline or stabilization, but after 2019, there was an upward trend, particularly in countries with middle SDI or higher. This suggests that, after 2019, falls due to reduced bone mass in the middle-aged population have contributed to an increase in health span loss (Figure 5A).

### 3.7 Predictive data

**Figure 6:**
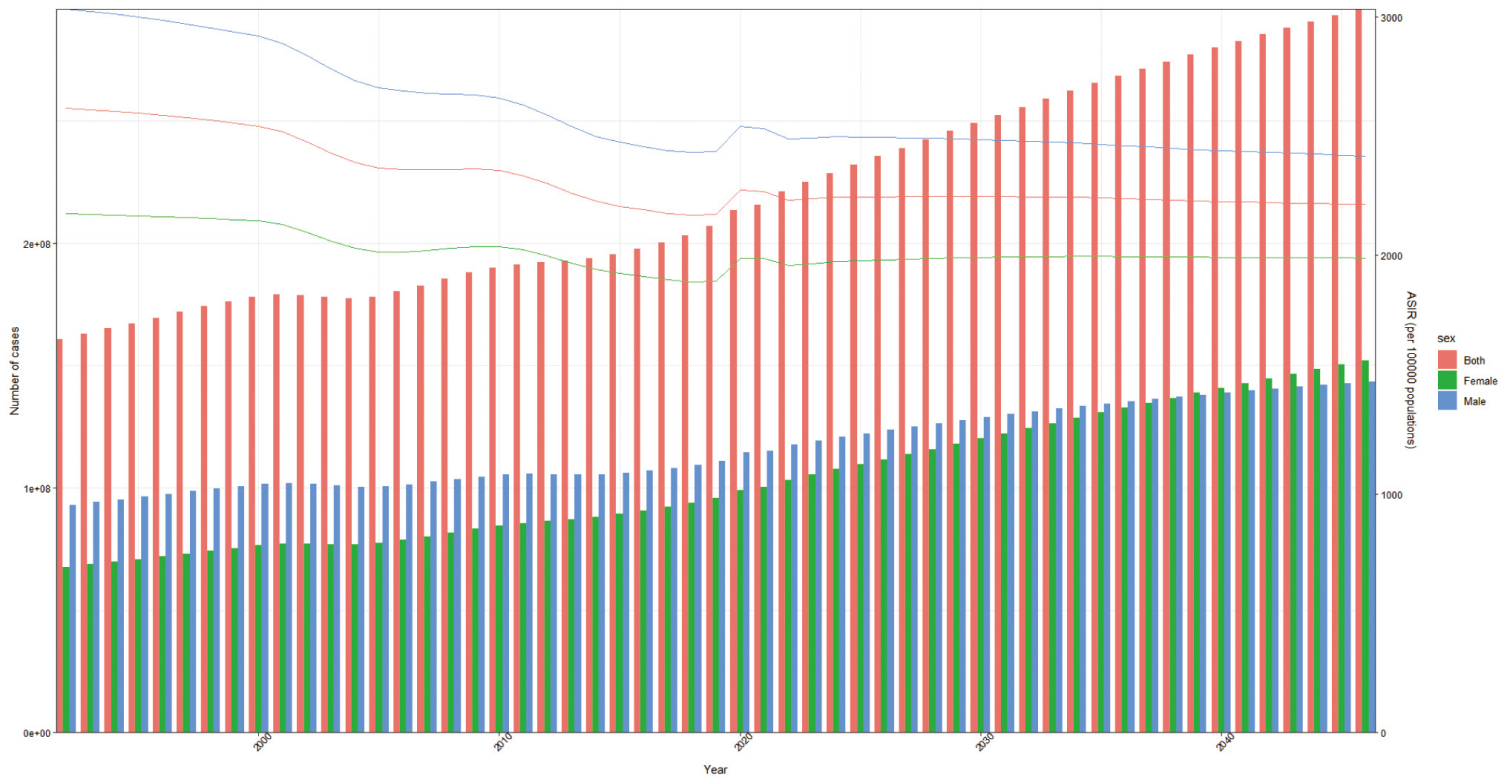
Fall burden projections to 2045, categorized by gender.

Based on estimated changes in population and incidence rates, although the **ASIR** continues to decline, the ongoing global population growth may affect the total number of cases. It is estimated that by 2045, nearly 300 million people worldwide will experience falls. Among the total fall cases in 2045, the number of predicted cases for women will exceed 150 million, while the predicted cases for men will surpass 140 million (Figure 6).

## 4 Discussion

Since the release of the GBD 2021 update, there has been a lack of comprehensive studies utilizing the latest data to assess falls. In this study, we compiled four key variables: incidence, prevalence, mortality, and DALYs. First, we summarized the trends in disease burden over the past decades, followed by an analysis of the distribution of falls due to low bone mass across various age groups and different SDI levels. Subsequently, we used the demographic projections from the GBD to forecast the future burden of falls over the coming decades, providing valuable insights for the formulation of related health policies.

From 1990 to 2021, our analysis reveals that over the past 32 years, the global burden of falls, including morbidity, incidence, mortality, and DALYs, has seen a significant increase, although age-standardized rates (ASR) have been on the decline. Additionally, the disease burden has increased with population aging. In 1990, the age-standardized incidence rate (ASIR) in low SDI regions was the lowest, while the age-standardized death rate (ASDR) was at an intermediate level. By 2021, the ASIR in low SDI regions remained the lowest, while the ASDR had reached its highest point. From a national and regional perspective, the number of cases in 2021 has nearly doubled compared to 2019. Our predictive model suggests that while the ASIR will gradually decline in the coming decades, the number of cases will continue to rise.

Falls-related injuries are negatively correlated with the level of education. Children under 5 years, women aged over 55, and men over 60 with an education level lower than primary school are identified as the most at-risk populations (15). Additionally, older age, female sex, a history of ischemic stroke, depression, rheumatoid arthritis, and kidney disease are significantly associated with an increased risk of falls (16–18). Furthermore, the use of prescription opioids is associated with an increased risk of severe fall events across all adult age groups, with the highest risk observed in individuals aged 85 and above (19). From 1990 to 2021, the continued global economic development and increasing education levels worldwide have contributed to a gradual decline in the global ASIR of falls. However, the other effect of economic development, including continuous population growth and aging, has led to an increase in the number of fall-related cases, a trend that is expected to continue over the next few decades (20).

In the broader context of a declining global ASIR, high SDI regions have consistently shown higher ASIRs, while low SDI regions, although maintaining a lower ASIR, had the highest ASDR in 2021. This discrepancy reflects differences in national healthcare responses and emergency capabilities. In the context of falls related to reduced bone mass, we observed a slight increase in DALYs across most regions, particularly in more developed countries, after 2019. This phenomenon was more pronounced among the middle-aged population (aged 40–59 years). This may be associated with the impact of the COVID-19 pandemic in 2019. With the onset of the pandemic, most countries implemented strict home quarantine measures, including stay-at-home orders, especially in middle- to high-income nations. In addition to older adults who were already less active or had limited mobility, these restrictions likely led to a marked reduction in physical activity among middle-aged individuals. The relationship between lack of exercise and decreased bone mineral density has been well-documented in several studies (21–23). The decline in physical activity among middle-aged adults likely resulted in reduced bone mass, consequently increasing DALYs attributable to falls associated with low bone mineral content (24–26). Therefore, insufficient physical activity leading to reduced bone mass can cause a range of adverse outcomes, it is essential to implement preventive measures in similar situations.

Some studies suggest that falls are becoming more prevalent among younger populations due to increased mobile phone usage and participation in risky activities (3). However, an analysis of the GBD data does not reveal a clear trend in this regard. Our findings show that the number of DALYs attributable to falls caused by reduced bone mass is higher in women than in men, particularly in individuals aged 50 and above. This disparity may be related to hormonal changes in women during menopause, which affect bone metabolism regulation.

For women, menopause is characterized by the cessation of estrogen production by the ovaries. Estrogen plays a crucial role in maintaining bone metabolism balance by inhibiting bone resorption and promoting bone formation, thus preserving bone density and strength. After estrogen levels decline, bone resorption accelerates significantly, while bone formation decreases, leading to reduced bone mass (27, 28). Moreover, estrogen inhibits the activity of osteoclasts. When estrogen is deficient, osteoclast activity increases, leading to more bone breakdown and a reduction in bone density (29, 30). Therefore, estrogen deficiency is a core pathological factor contributing to the high incidence of osteoporosis in postmenopausal women. This accelerated bone loss likely explains the higher DALYs in women compared to men. While bone density naturally declines with age, women experience a more rapid rate of bone loss after menopause due to the significant reduction in estrogen levels (31, 32). Similarly, in studies of falls from all causes, the age-standardized incidence rate (ASIR) of falls in women gradually surpassed that of men after the age range of 55–59, with an increasing gap. Additionally, in older individuals with osteoporosis, the risk of falls increases due to impaired standing ability, compromised dynamic balance, and reduced walking speed (33).

As individuals age, their physical function, balance, and flexibility decline, leading to an increased incidence of falls. By 2050, one-fifth of the global population will be aged 60 or older, with 19% of people aged 80 or above (34). Therefore, over the coming decades, as the population continues to grow and age, the number of falls will keep increasing, posing a major challenge to global health systems. This necessitates targeted prevention and control strategies that address risk factors and are tailored to specific regions (particularly those with developmental disparities). However, this situation is not inevitable. Studies have shown that even in older populations, strength and balance training can activate physiological compensatory mechanisms, improve balance function, and effectively reduce the risk of falls (35). In addition, quitting smoking and limiting alcohol consumption, together with increased sunlight exposure, can help maintain calcium absorption and vitamin D metabolism, thereby protecting bone health. Engaging in moderate and regular physical activity—such as weight-bearing exercises, resistance training, and balance or flexibility exercises—can effectively stimulate bone remodeling, improve muscle strength, and enhance body coordination. These activities play a crucial role in preventing both osteoporosis and falls (36, 37). In the future, customized interventions for middle-aged and older individuals can be developed, improving the effectiveness of fall prevention measures.

## Limitations

(1) There are significant differences in healthcare system development across countries. As noted by Vincent Wai-Sun Wong et al., the use of different diagnostic methods may lead to varying rates of disease diagnosis (38), which can result in discrepancies in disease diagnosis and management. Such differences may increase the likelihood of misdiagnosis or underdiagnosis, potentially introducing bias into clinical outcomes and epidemiological data. However, this does not affect the global trend analysis of the disease.
(2) Due to limitations in data availability, it was not possible to directly obtain specific values for the disease. As a result, this study relied on estimates for analysis, which may affect the precision and reliability of the results.
(3) Although a robust predictive model was employed, certain uncertainties remain. The nordpred model assumes that future trends will follow a linear or near-linear continuation of past patterns. However, the assumptions of trend continuation and gradual attenuation may not fully capture the potential impacts of future social, environmental, or health policy changes—for example, sudden events such as the COVID-19 pandemic in 2019. Moreover, the decomposition of age, period, and cohort effects is subject to inherent identifiability constraints, and limitations in data quality may further affect the reliability of long-term projections.

## Data Availability

The original contributions presented in the study are included in the article/Supplementary material, further inquiries can be directed to the corresponding author.
