# Supplementary material for: Global, regional, and national burden of falls from 1990 to 2021: a comprehensive analysis with a focus on falls attributable to reduced bone mass and projections to 2045
Source: Front Public Health. 2025 Nov 11;13:1695026. doi: 10.3389/fpubh.2025.1695026 (PMC12646055; doi:10.3389/fpubh.2025.1695026)
Supplement: Supplementary file 1 [file Table_1.docx]

Table 1

Prevalence, Incidence, Deaths and DALYs cases and the age-standardized rates of Falls by 17 GBD regions.

| location | Count1990（Prevalence） | ASR1990（Prevalence） | Count2021（Prevalence） | ASR2021（Prevalence） | Count1990（Incidence） | ASR1990（Incidence） | Count2021（Incidence） | ASR2021（Incidence） | Count1990（Deaths） | ASR1990（Deaths） | Count2021（Deaths） | ASR2021（Deaths） | Count1990（DALYs） | ASR1990（DALYs） | Count2021（DALYs） | ASR2021（DALYs） |
| --- | --- | --- | --- | --- | --- | --- | --- | --- | --- | --- | --- | --- | --- | --- | --- | --- |
| Andean Latin America | 1149644.7 (975467.4-1380053.9) | 3912.5 (3366.2-4620.5) | 3115838.1 (2670027.6-3653806) | 4808.7 (4134-5624.4) | 659413.5 (571439-765366.2) | 1621.5 (1437.1-1832.6) | 1393528.8 (1238555.6-1573854.8) | 2074.6 (1846-2338.8) | 1789.5 (1578.5-2057.2) | 7.4 (6.5-8.5) | 3878.2 (3107-4698.4) | 6.5 (5.2-7.9) | 129249.2 (110200.8-151241.9) | 425.5 (364.4-501.7) | 253204.8 (207671.3-310781.5) | 395.5 (324.2-485.4) |
| Australasia | 2661537.8 (2248164.8-3234673.5) | 12159.1 (10264-14835.4) | 5272295.6 (4572918.3-6216234.1) | 12862.8 (10938.8-15735.4) | 1237056.1 (1048956.7-1470394.4) | 6385.1 (5338.4-7744.9) | 2179050 (1949663.4-2457998.8) | 6948.3 (5892.6-8323) | 1163.8 (1049.5-1237.2) | 5.5 (4.9-5.9) | 5144.2 (4282.5-5646.5) | 8.2 (6.9-8.9) | 133451 (96577.4-177369.7) | 611.7 (443.6-813.5) | 303091.3 (232538.5-389512.4) | 680.9 (505-900.9) |
| Caribbean | 1100323 (945326-1301810.1) | 3682.8 (3204.3-4306.6) | 2195604.2 (1928003.8-2528386) | 4223.7 (3701.2-4883.4) | 536342.8 (471207.9-620429.6) | 1564.5 (1399.9-1775.8) | 897677 (818367.7-989722.7) | 1832.9 (1653.9-2038.7) | 2256 (2109.6-2373) | 9.7 (9.1-10.2) | 5651.8 (4981.5-6258.4) | 10.1 (9-11.2) | 105280.4 (89854.8-123710.7) | 368 (317.8-429) | 200756.9 (170384.8-237412.2) | 381.3 (322.7-451.6) |
| Central Asia | 4484409.5 (3779804.4-5414187.8) | 7541.4 (6412.3-8968.9) | 5512448 (4671037.8-6482716.6) | 5871 (4992.8-6903.1) | 2363458.9 (2116320.3-2626771.3) | 3137.8 (2838.1-3463.8) | 2384289.7 (2143532.1-2651368.2) | 2494.8 (2237.4-2781.3) | 3736.6 (3533.1-3913.5) | 6 (5.7-6.2) | 3229.6 (2878.1-3595.8) | 3.7 (3.3-4.1) | 405783.5 (347905.9-477894.5) | 619.5 (524.3-738.7) | 380631.4 (310934.9-468164.4) | 402.6 (328.9-495.8) |
| Central Europe | 17932479.9 (15453351-20607187.4) | 12987.3 (11202.5-14986.1) | 17918196.5 (15518558.1-20377774.7) | 10902 (9372.6-12682.6) | 7249154.3 (6554578-8013853.2) | 5795.9 (5217-6453.4) | 5791945.9 (5336054.5-6316125.6) | 4899.2 (4362.3-5540.1) | 18708.3 (17885.7-19366.2) | 15 (14.2-15.6) | 16036.4 (14520.8-17152) | 7.6 (6.9-8.1) | 1283759.8 (1047555.7-1564509.9) | 952.5 (780.9-1155.5) | 1078481.9 (841661-1344160.5) | 632.9 (490.3-802) |
| Central Latin America | 11576156.8 (9558288-14453607.2) | 9123.1 (7724-11011.4) | 15922886.5 (13501558.7-19277981.1) | 6144.3 (5222.1-7431.5) | 6680891.6 (5634229.2-7905612.4) | 3787.9 (3292.4-4371.3) | 6537780 (5715234.6-7581210.2) | 2619.2 (2268.8-3048.4) | 10276 (10014.7-10483.7) | 11.2 (10.8-11.5) | 13106.5 (11766-14448.4) | 5.4 (4.8-5.9) | 931910.6 (774937.4-1116779.1) | 739.1 (616.7-881.5) | 1043910.9 (833958.6-1282357.7) | 407.3 (326.9-499.6) |
| Central Sub-Saharan Africa | 680910.5 (572851.5-826878.6) | 1944.3 (1692.7-2263.3) | 1864031.3 (1577448.9-2246869.8) | 2122.6 (1863.8-2444.6) | 439156.5 (366528-526079.8) | 823.9 (720.5-944.5) | 1187242.1 (994837.9-1402477) | 925.3 (822.8-1040.6) | 2162.8 (1692.3-2899.9) | 10.8 (8.5-14.4) | 4751.7 (3439.7-6836.6) | 10.5 (7.8-15.1) | 127888 (99646.2-178301.3) | 360 (298.1-456) | 269093.2 (211937-352053.7) | 338.2 (266.6-439.9) |
| East Asia | 54159989 (46329179.7-64236456.1) | 5063.2 (4388-5916.5) | 113238528.1 (98639456.8-129396253) | 5978.2 (5205.2-6813.7) | 25222205.1 (21549079-29735971.9) | 2102.2 (1822.1-2437.3) | 40322894.1 (36270313.8-44856386.4) | 2684.5 (2397.6-3026.9) | 75591.3 (64284.5-99592.2) | 10 (8.7-12.9) | 146150.6 (95163.5-187800.6) | 8.5 (5.5-10.9) | 5984239 (4952395.3-7436381.6) | 567.4 (474.9-701.6) | 8488967.7 (6536064.8-10672804.8) | 474.9 (370.3-590.1) |
| Eastern Europe | 34743637.5 (28894350-40345145) | 13621.8 (11314.6-15740.7) | 33613088.4 (28141783.8-39404136.8) | 12160 (10166.4-14212.1) | 13295015.7 (11420526.8-15524879.5) | 5998.1 (5128.7-6996.3) | 10654143 (9484661.9-12013114.3) | 5454.4 (4793.4-6226.1) | 15570.8 (15239.9-15836.5) | 6.5 (6.3-6.6) | 17601.3 (16272.9-18827.2) | 5.9 (5.5-6.3) | 2087621.6 (1645135.6-2625405.1) | 840.8 (667.8-1051.7) | 1897353.2 (1482154.6-2447510.7) | 703.6 (551.8-893.5) |
| Eastern Sub-Saharan Africa | 2481891.6 (2106271.9-3009729.9) | 2074 (1800.9-2416.8) | 5738601.3 (4861706.8-6889901.9) | 2093.4 (1834.9-2419.8) | 1643295.2 (1342263.7-1993808.9) | 871.8 (752.2-1013.2) | 3642715.8 (3056341.3-4345605.2) | 911.8 (808.3-1037) | 11204.9 (8863.2-13045.5) | 17 (14.4-20.1) | 21218.1 (17892.7-24921.3) | 15 (12.8-17.7) | 559382.6 (439869.6-669004.6) | 482.3 (411.2-556) | 1010808.3 (851549.2-1182108.5) | 416.2 (357.9-481.4) |
| Global | 326901030.2 (280676231-382950669.2) | 7279.6 (6310.5-8385.2) | 540882882.2 (472963219.2-615109903.6) | 6455.4 (5652.6-7337.1) | 155954194.5 (136202891-179718954.7) | 3002.3 (2665.3-3411.4) | 215566471.5 (195267830-238982230.1) | 2702 (2444.5-3000.4) | 407767.8 (357240.4-450139.6) | 10.9 (9.7-11.8) | 802802.6 (681873.5-874337.7) | 9.9 (8.4-10.8) | 29408340.8 (24558088.2-35187652.1) | 643.1 (539.5-767.6) | 43804122.6 (35938837.2-52811147.3) | 531.3 (436.9-639.1) |
| High-income Asia Pacific | 17401418.9 (14971928.2-20159045.2) | 8983.1 (7736.2-10437.7) | 23243541.3 (20439779.7-26313591.9) | 7349.3 (6357.9-8492.9) | 8056281.2 (7055970.3-9229966.8) | 4722.3 (4133.6-5431) | 7357241.7 (6680230.9-8115924.3) | 3752.2 (3252.6-4347.5) | 8360.7 (7597.9-9120.5) | 4.6 (4.2-5.1) | 21619.6 (17682.1-24289.7) | 3.8 (3.3-4.3) | 1029762 (801797.8-1323498.7) | 541 (424.1-691.5) | 1345484 (1031164.5-1726656.1) | 402.2 (302-523) |
| High-income North America | 24337704.9 (21129304.9-27740893) | 7466.7 (6452.3-8542.4) | 42740101.6 (37754436.4-48099875.3) | 7243.7 (6430.1-8138.8) | 10740466.5 (9305546.6-12412221.8) | 3716.2 (3208.7-4334.9) | 16454980.8 (15002506.2-18172212.3) | 3463.2 (3123.5-3846.2) | 15802.3 (14121.3-16628) | 4.5 (4-4.7) | 58297.9 (49792.2-62768.8) | 8.2 (7.1-8.8) | 1386309.1 (1059636.3-1774055) | 424.2 (323.4-544.2) | 2819344.5 (2264787.5-3553753.1) | 469.1 (373.9-590.4) |
| High-middle SDI | 94706400.8 (80678459-109960970.5) | 9068.4 (7759.6-10467.2) | 134351429.5 (116787547.8-153056484.4) | 7952.3 (6888.2-9131.7) | 40200945.1 (35126899.4-46287968.4) | 3764.8 (3311-4309.7) | 46475844 (42053798-51668557.1) | 3567.9 (3186.3-4037) | 75471.6 (69309.6-84134.7) | 8.7 (7.9-9.6) | 125210 (97061-147860.3) | 7.1 (5.5-8.3) | 6898871.3 (5596022.4-8432583.7) | 676.5 (552.2-824.4) | 8524638.6 (6774352.7-10781811.2) | 513.5 (407.5-644) |
| High SDI | 99644048.8 (86502153-114227323.2) | 9831.4 (8520.6-11293.3) | 151266875.7 (133520189-169573730.8) | 9086.6 (7948.3-10367.3) | 43506474.4 (38827743.4-49176850) | 4895.4 (4315.3-5611.3) | 56011530.2 (51533089.2-60975194.5) | 4533.9 (4026.5-5130.2) | 81191.7 (74766.8-84617.2) | 7.8 (7.2-8.1) | 175612.9 (150396.8-189642.4) | 7.3 (6.4-7.8) | 6169362.7 (4822132-7762556) | 611.4 (478.3-769.2) | 9423764.4 (7418025-11863731.3) | 536.5 (413.9-683.9) |
| Low-middle SDI | 44556612.9 (38122377-53067954.3) | 5348.2 (4653.8-6251.3) | 80360254.8 (69954951.3-92281754.4) | 4908.9 (4325.8-5565.4) | 25044130.2 (21437762.9-29290599.6) | 2239.9 (1969-2555.1) | 38334227.9 (34208776.9-42915812.4) | 2094.9 (1890.4-2313.2) | 96693.8 (76477.7-110501.2) | 16.4 (13.6-19) | 197589 (168854.6-219803.2) | 15.8 (13.5-17.7) | 5881534.9 (4818476.6-6937683.5) | 694.9 (589-798.4) | 9409130 (8037525.4-10894882.9) | 604.1 (519-690.8) |
| Low SDI | 11254038 (9610684.4-13471455.5) | 3473.3 (3039.3-4052.5) | 24775175.8 (21454075-29151926.8) | 3473.9 (3055.8-3954.5) | 6804880 (5740100.3-7977712.6) | 1426 (1257.8-1620.3) | 14482250.2 (12586183-16584663.1) | 1456.5 (1312.7-1612.4) | 38775.5 (30558-45805.7) | 18.5 (15.8-21.7) | 75441.7 (62892.6-87667.6) | 17.4 (14.7-20.3) | 2123255.8 (1658944-2566598) | 622.7 (529.2-719.9) | 3771452.6 (3166176.2-4394861.4) | 551.7 (474.1-631.2) |
| Middle SDI | 76330709.7 (65266590.4-91007183.6) | 5493.1 (4761.8-6463.2) | 149633430 (130369545.9-171006274.8) | 5669.8 (4947.9-6487) | 40222235.8 (34549746.6-47081020.4) | 2319.7 (2028.1-2669.8) | 60079578.2 (53913893.7-66900142.9) | 2466 (2213-2751.4) | 115139 (99080.3-128957.9) | 11.8 (10.1-12.8) | 228295.9 (178914.1-259593.8) | 9.9 (7.7-11.2) | 8303426.8 (6980456.6-9804568.1) | 598.6 (509.3-701.5) | 12639313.4 (10398866.8-15132593.5) | 495.7 (409-589.9) |
| North Africa and Middle East | 11463833.3 (9670322-13803690.6) | 4537.6 (3874.4-5330.1) | 30322049.8 (25687815.9-35029183.5) | 5234 (4476-6011.5) | 6937401.3 (5994765.5-8108164.9) | 1911.8 (1692.7-2192.1) | 14287405.9 (12707681.6-16185936.5) | 2281.7 (2042.5-2564.7) | 15051.4 (12020.8-18835.9) | 7.7 (6.3-9.3) | 23404.4 (20141.7-27599.2) | 5.7 (4.8-6.7) | 1286127.2 (1003703-1613947.7) | 451.2 (363.1-550.6) | 2165158 (1782266.4-2689369.6) | 385.6 (318.8-475) |
| Oceania | 134966.1 (114080.4-160727.4) | 3059.8 (2647.8-3536.5) | 417433.8 (355833.5-495666.5) | 4033 (3533.5-4675) | 80710 (68473.9-95165.8) | 1306.2 (1155.3-1487) | 227214.3 (203624-253155.8) | 1763.2 (1608.3-1932.3) | 218 (154.9-286.5) | 7.9 (5.4-10.6) | 559.7 (340.1-793.6) | 7.9 (4.8-11.3) | 16279.8 (13237.6-19722.7) | 362.4 (287.9-445.3) | 43751.2 (33852.6-56534.8) | 421.6 (321.1-542) |
| South Asia | 48428174.4 (41524201.2-57759209.3) | 6265.6 (5438.7-7246) | 97274936.1 (85051478.9-110996540.9) | 6000.7 (5289.2-6785) | 26869032.3 (23060972.5-31292692.1) | 2668.6 (2329.3-3031.1) | 46081178.5 (41202495.7-51175978.3) | 2629.4 (2361.4-2905.5) | 117025.8 (86891.7-136673.8) | 22.8 (17.5-26.9) | 262196.1 (214422.7-292785.2) | 21.2 (17.3-23.8) | 6647830.3 (5325568.9-7775440.4) | 874 (721.1-1009.7) | 11646796.3 (9746016-13595118.3) | 764.8 (641.9-881.3) |
| Southeast Asia | 12252850.1 (10579536.7-14334144.8) | 3562.4 (3118.8-4074.1) | 26289889.7 (23039108.3-30048014.9) | 3824.4 (3371.6-4329.1) | 6821008.5 (5931736.3-7898107.4) | 1520.2 (1348.1-1715.2) | 11390039 (10294207.5-12554639.6) | 1675.7 (1517.5-1840.2) | 30963.6 (25789.2-35324.9) | 12.6 (9.6-14.8) | 58275.7 (46640.1-66398.6) | 10.4 (8-11.8) | 1769006.4 (1487576.2-2074530.1) | 513.7 (443.6-590.2) | 2970332 (2526498-3453428.1) | 449.4 (381.7-521.5) |
| Southern Latin America | 3063961.2 (2652001.2-3611254.2) | 6478.8 (5630.6-7605.7) | 5505391.3 (4767240.6-6504803.6) | 7068.8 (6088.5-8439) | 1611714.1 (1400211.4-1859286.7) | 3234.2 (2826.4-3712.1) | 2344702.4 (2057696.3-2706862.1) | 3567.5 (3097.4-4179.8) | 2421.8 (2283.2-2526.5) | 5.8 (5.4-6.1) | 3533.9 (3156.9-3754.1) | 4 (3.6-4.2) | 213431 (169783.9-263748) | 456.6 (364.6-564) | 314359.5 (239250.6-403213.1) | 398.9 (302.6-515.7) |
| Southern Sub-Saharan Africa | 873322.3 (738137-1024003) | 2273.4 (1945.8-2628.2) | 1395494.9 (1183872.1-1617233.1) | 1920.3 (1648.2-2202.5) | 509592.7 (418245.3-624247.5) | 950.1 (800.1-1131.5) | 682101.9 (577909.1-814158.4) | 836 (716.8-987.4) | 1092.8 (912.6-1266.1) | 3.8 (3-4.4) | 1732.7 (1486.1-2171.3) | 3 (2.6-3.8) | 83639 (71077.2-99915.4) | 214.7 (181.4-257.9) | 127179.3 (105890.7-156400.4) | 177.3 (148.4-217.1) |
| Tropical Latin America | 14231525.2 (11952600.5-17050681.5) | 11253.1 (9478.8-13224.7) | 21231348 (18214823-24687827) | 8459.3 (7267.2-9897.8) | 7561717 (6429709.8-8988741.1) | 4656.8 (4001.3-5486.6) | 8098424.2 (7167874.6-9190557) | 3605.6 (3161.3-4128.1) | 7876 (7543-8101.9) | 8.5 (7.9-8.8) | 21920.2 (19620.2-23307.5) | 9 (8-9.6) | 950783.7 (765052-1177535.8) | 758.5 (610.2-932.8) | 1412652.4 (1138856.8-1735970.7) | 565.4 (456.6-693.6) |
| Western Europe | 60846785.7 (53159778.8-69486036.6) | 12633.4 (10947.6-14630.2) | 80479012.4 (71116706.4-91286439.5) | 11979.7 (10359.8-14084.4) | 25639286.6 (23113206.4-28724157.7) | 6629.1 (5827.5-7580.9) | 28768356.5 (26336964.8-31465406.5) | 6210.5 (5390.1-7183.3) | 54532.2 (49614.1-57205.5) | 9.9 (9-10.4) | 91136.8 (76595.2-98623.4) | 7.8 (6.8-8.4) | 3689428.1 (2862716.3-4658615.7) | 754.9 (582.1-956.3) | 4826638.7 (3763475.7-6100831.5) | 654.2 (491.8-844.3) |
| Western Sub-Saharan Africa | 2895507.7 (2451415.5-3524603.9) | 2263.3 (1959.4-2649.5) | 7592164.9 (6455418.5-9138268.9) | 2430.4 (2119.7-2815.4) | 1800994.3 (1477408.1-2188032.4) | 955.2 (825.4-1107.8) | 4883560.1 (4091365.4-5745329.6) | 1064.2 (946.2-1197) | 11963.1 (10186.5-14452.9) | 14.7 (12.4-17.8) | 23357 (19090.6-27588.2) | 13.4 (11.4-15.8) | 587178.6 (490296.2-701366.9) | 452 (392.7-529.7) | 1206127.2 (1004749.9-1411992) | 407.4 (341.1-472.8) |
